# Supplementary material for: Primate homologs of mouse cortico-striatal circuits
Source: eLife. 2020 Apr 16;9:e53680. doi: 10.7554/eLife.53680 (PMC7162658; doi:10.7554/eLife.53680)
Supplement: Supplementary file 1. [file elife-53680-supp1.docx]

**Supplemental Table 1**: Mouse target regions used for connectivity fingerprint matching. RsfMRI timeseries were extracted from 3x3x3 voxel cubes drawn around the x,y,z co-ordinates (Franklin and Paxinos, 2019). References are provided to support homologies across species.

|  | **Human/Primate Label** | **Mouse label** | **Franklin & Paxinos** | | | **Reference** |
| --- | --- | --- | --- | --- | --- | --- |
|  |  |  | **x** | **y** | **z** |  |
| **CINGULATE** | Area 25 | IL | 0.2 | 2.75 | 1.54 | (Heilbronner et al., 2016; Vogt and Paxinos, 2014) |
|  | Area 32pl | PL | 0.25 | 2 | 2.22 | (Heilbronner et al., 2016; Vogt and Paxinos, 2014) |
|  | Area 24 | CG1+2 | 0.15 | 2 | 0.98 | (Heilbronner et al., 2016; Vogt and Paxinos, 2014) |
|  | Retrosplenial cortex | Retrosplenial area, ventral + dorsal part | 1.25 | 1 | -1.94 | (Vogt and Paxinos, 2014) |
| **ORBITALFRONTAL** | Area 13 | Orbitofrontal lateral | 1.25 | 2.5 | 2.46 | (Wise, 2008) |
| **AMYG** | Basolateral Amygdala | Basolateral amygdalar nucleus | 2.9 | 4.7 | -2.06 | (Heilbronner et al., 2016) |
| **HIPPO** | Anterior Hippocampus | Ventral Hippocampus | 2.25 | 2.5 | -3.6 | (Strange et al., 2014) |
|  | Posterior Hippocampus | Dorsal Hippocampus | 0.5 | 2.1 | -1.58 | (Strange et al., 2014) |
| **MOTOR** | M1 | Primary motor area | 1.75 | 1.5 | 1.42 | (Balleine and apos Doherty, 2009) |
|  | S1 | average of somatosensory areas | 2.5 | 1.5 | 0.26 | (Balleine and apos Doherty, 2009) |
|  | S2 (OP1) | Supplemental somatosensory area | 3.5 | 3 | -0.22 | (Balleine and apos Doherty, 2009) |
| **TEMPORAL** | TPJp | Temporal Association Area | 4.1 | 3.1 | -2.8 | (Grandjean et al., 2019; Zerbi et al., 2015) |

**Supplemental Table 2**: Macaque target regions used for connectivity fingerprint matching. RsfMRI timeseries were extracted from 3x3x3 voxel cubes drawn around the x,y,z co-ordinates (McLaren et al., 2009). References are provided to support homologies across species.

|  | **Label** | **F99 space (McLaren)** | | | **Reference** |
| --- | --- | --- | --- | --- | --- |
|  |  | **x** | **y** | **z** |  |
| **CINGULATE** | *Area 25* | 2.25 | 10.5 | 1.8 | (Neubert et al., 2015; Paxinos et al., 2009) |
|  | *Area 32* | 2.25 | 14.9 | 7.8 | (Neubert et al., 2015; Paxinos et al., 2009) |
|  | Area 24 | 2.25 | 7.5 | 15.3 | (Neubert et al., 2015; Paxinos et al., 2009) |
|  | Retrosplenial cortex | 2.25 | -22.3 | 4.8 | (Margulies et al., 2009) |
| **ORBITALFRONTAL** | Area 13L | 9.7 | 11.9 | 4.8 | (Neubert et al., 2015; Paxinos et al., 2009) |
| **AMYG** | Basolateral Amygdala | 9.7 | 0 | -10.2 | (Paxinos et al., 2009) |
| **HIPPO** | Anterior Hippocampus | 14.1 | -8.5 | -11.7 | (Paxinos et al., 2009) |
|  | Posterior Hippocampus | 15.6 | -16.35 | -5.72 | (Paxinos et al., 2009) |
| **MOTOR** | M1 | 14 | -9 | 16.77 | (Neubert et al., 2015) |
|  | S1 | 12.65 | -13.4 | 18.3 | (Neubert et al., 2015) |
|  | S2 (OP1) | 20 | -16.3 | 10.77 | (Neubert et al., 2015) |
| **TEMPORAL** | TPJp | 21.5 | -13.3 | -2.72 | (Mars et al., 2013) |
| **DLPFC** | Area 9/46d | 12.65 | 11.9 | 13.8 | (Paxinos et al., 2009) |
|  | Area 9/46v | 15.6 | 11.9 | 9.28 | (Paxinos et al., 2009) |
| **VLPFC** | *Area 44* | 20 | 6 | 7.77 | (Neubert et al., 2014) |
| **FRONTAL POLE** | *Area 10m* | 0.7 | 23.8 | 7.8 | (Neubert et al., 2014) |
| **MEDIAL PFC** | SMA | 2.2 | -3 | 21.3 | (Neubert et al., 2015) |

**Supplemental Table 3**: Human target regions used for connectivity fingerprint matching. RsfMRI timeseries were extracted from 3x3x3 voxel cubes drawn around the x,y,z co-ordinates in MNI space. References are provided to support homologies across species.

|  |  | **Label** | **MNI** | | | **Reference** |
| --- | --- | --- | --- | --- | --- | --- |
|  |  |  | **x** | **y** | **z** |  |
| **CINGULATE** | **1** | Area 25 | 4 | 14 | -10 | (Neubert et al., 2015) |
|  | **2** | Area 32pl | 2 | 40 | 2 | (Neubert et al., 2015) |
|  | **3** | Area 24 | 2 | 18 | 28 | (Neubert et al., 2015) |
|  | **4** | Retrosplenial cortex | 2 | -44 | 22 | (Neubert et al., 2015) |
| **ORBITALFRONTAL** | **5** | Area 13 | 18 | 24 | -22 | (Neubert et al., 2015) |
| **AMYG** | **6** | Basolateral Amygdala | 26 | -2 | -18 | (Amunts et al., 2005) |
| **HIPPO** | **7** | Anterior Hippocampus | 26 | -16 | -20 | (Amunts et al., 2005) |
|  | **8** | Posterior Hippocampus | 32 | -34 | -6 | (Amunts et al., 2005) |
| **MOTOR** | **9** | M1 | 40 | -20 | 58 | (Mayka et al., 2006; Neubert et al., 2015) |
|  | **10** | S1 | 44 | -32 | 58 | (Mayka et al., 2006; Neubert et al., 2015) |
|  | **11** | S2 (OP1) | 56 | -22 | 20 | (Eickhoff et al., 2010; Neubert et al., 2015) |
| **TEMPORAL** | **12** | TPJp | 54 | -60 | 28 | (Mars et al., 2013) |
| **DLPFC** | **13** | Area 9/46d | 28 | 40 | 32 | (Sallet et al., 2013) |
|  | **14** | Area 9/46v | 44 | 26 | 36 | (Sallet et al., 2013) |
| **VLPFC** | **15** | Area 44d | 56 | 18 | 24 | (Neubert et al., 2014) |
| **FRONTAL POLE** | **16** | Area FPm | 6 | 58 | 0 | (Neubert et al., 2015) |
| **MEDIAL PFC** | **17** | SMA | 6 | -10 | 60 | (Neubert et al., 2015) |

**Supplemental Table 4**: Regions showing significant connectivity (voxel-level Z>3.1, cluster-level p<0.05 FDR) with the human-mouse mCP map. K indicates cluster size in voxel number (each voxel is 2x2x2mm). X, Y, Z co-ordinates are in MNI space. Cortical localization used the Harvard-Oxford probabilistic atlas, Jülich atlas, and connectivity-based parcellation atlases provided in FSLEYES. Striatal localization used the atlases of Pauli et al (Pauli et al., 2016), Choi et al (Choi et al., 2012), and Tziortzi et al (Tziortzi et al., 2014). Cerebellar localization used the probabilistic atlas of Diedrichsen et al (Diedrichsen et al., 2009).

| **Label** | **K** | **x** | **y** | **z** | **T** | **Harvard Oxford** | **Juelich** | **Connectivity** | **Diedrichsen** | **Pauli** | **Choi** |
| --- | --- | --- | --- | --- | --- | --- | --- | --- | --- | --- | --- |
| Frontal Pole | 137 | 30 | 56 | -8 | 10.12 | Frontal Pole (54%) | FP1 (62%) | Area 47m (76.1%) |  |  |  |
| Middle Frontal Gyrus | 870 | 34 | 56 | 22 | 11.85 | Frontol Pole (84%) |  | Area 46 (54.6%) |  |  |  |
| Middle Cingulate Cortex | 1080 | 8 | 18 | 32 | 11.79 | Cingulate Cortex, anterior division (54%) |  | RCZa (62.5%) |  |  |  |
| Insula | 434 | 38 | 16 | 0 | 9.92 | Insular Cortex (84%) |  |  |  |  |  |
| Caudate Nucleus | 2057 | 12 | 16 | 6 | 27.36 | Caudate (98.7%) |  | Executive (73.1%) |  | Action Value | DMN |
| Nacc | 2057 | 10 | 14 | -6 | 31 | Nacc (93.7%) |  | Limbic (61.5%) |  | Stimulus value | Limbic |
| Putamen | 2057 | 26 | 6 | 4 | 20.48 | Putamen (99%) |  | Executive (69.2%) |  | Social/Language | Ventral Attention |
| Hippocampus | 17 | 26 | -22 | -10 | 8.33 | Hippocampus (42.7%) |  |  |  |  |  |
| Cerebellum | 671 | 32 | -36 | -34 | 12.94 |  |  |  | Lobule HVI (60%) |  |  |
| Posterior Cingulate Cortex | 148 | 4 | -38 | 24 | 9.26 | Cingulate Cortex, posterior division (52%) |  | area 23ab (38.7%) |  |  |  |
| Supramarginal gyrus | 505 | 48 | -42 | 42 | 9.55 | Supermarginal Gyrus, posterior division (32%) | hIP2 (34%) | IPLC (87.5%) |  |  |  |
| Cerebellum | 46 | 26 | -48 | -46 | 8.37 |  |  |  | Lobule VIIIb(26%) |  |  |
| Precuneus | 239 | 12 | -76 | 46 | 9.02 | Lateral Occipital Cortex (36%) |  | SPLE (75%) |  |  |  |
| Occipital Pole | 964 | 26 | -100 | -4 | 11.31 | Occipital Pole (56%) | hOc1/V1 (42%) |  |  |  |  |

**Supplemental Table 5**: Regions showing significant connectivity (voxel-level Z>3.1, cluster-level p<0.05 FDR) with the human-mouse NAcc map. K indicates cluster size in voxel number (each voxel is 2x2x2mm). X, Y, Z co-ordinates are in MNI space. Cortical localization used the Harvard-Oxford probabilistic atlas, Jülich atlas, and connectivity-based parcellation atlases provided in FSLEYES. Striatal localization used the atlases of Pauli et al (Pauli et al., 2016), Choi et al (Choi et al., 2012), and Tziortzi et al (Tziortzi et al., 2014). Cerebellar localization used the probabilistic atlas of Diedrichsen et al (Diedrichsen et al., 2009).

| **Label** | **K** | **x** | **y** | **z** | **T** | **Harvard Oxford** | **Juelich** | **Connectivity** | **Diedrichsen** | **Pauli** | **Choi** |
| --- | --- | --- | --- | --- | --- | --- | --- | --- | --- | --- | --- |
| Anterior Cingulate Cortex | 15 | 10 | 40 | 8 | 7.95 | Cingulate Cortex, anterior division (46%) |  | Area 32pl (47.1%) |  |  |  |
| Middle Cingulate Cortex | 34 | 4 | 34 | 30 | 7.48 | Paracingulate Cortex (71%) |  | Area 32d (63%) |  |  |  |
| Nacc | 1423 | 10 | 12 | -6 | 53.29 | Nacc (92.3%) |  | Limbic (65.4%) |  | Stimulus value | Limbic |
| Cerebellum | 41 | 4 | -50 | -36 | 8.43 |  |  |  | Lobule IX (45%) |  |  |
| Cerebellum | 35 | 46 | -66 | -26 | 7.35 |  |  |  | Crus I (97%) |  |  |
| Occipital Pole | 61 | 26 | -102 | -4 | 7.44 | Occipital Pole (56%) | hOc1/V1 (42%) |  |  |  |  |

**Supplemental Table 6**: Regions showing significant connectivity (voxel-level Z>3.1, cluster-level p<0.05 FDR) with the human-mouse lCP map. K indicates cluster size in voxel number (each voxel is 2x2x2mm). X, Y, Z co-ordinates are in MNI space. Cortical localization used the Harvard-Oxford probabilistic atlas, Jülich atlas (* Area 6 probabilities were taken from the Jülich atlas in FSLEYES), and connectivity-based parcellation atlases provided in FSLEYES. Striatal localization used the atlases of Pauli et al (Pauli et al., 2016), Choi et al (Choi et al., 2012), and Tziortzi et al (Tziortzi et al., 2014). Cerebellar localization used the probabilistic atlas of Diedrichsen et al (Diedrichsen et al., 2009).

| **Label** | **K** | **x** | **y** | **z** | **T** | **Harvard Oxford** | **Juelich** | **Connectivity** | **Diedrichsen** | **Pauli** | **Choi** |
| --- | --- | --- | --- | --- | --- | --- | --- | --- | --- | --- | --- |
| Middle Frontal Gyrus | 79 | 32 | 44 | 26 | 12.36 | Frontal Pole (83%) |  | Area 9/46d (55.8%) |  |  |  |
| Posterior Medial Frontal | 525 | 2 | 6 | 58 | 13.44 | SMA (41%) | Area 6 (65%)* | SMA (36.2%) |  |  |  |
| Precentral Gyrus | 17 | 60 | 2 | 26 | 11.49 | Precentral Gyrus (60%) | Area 6 (32%)* |  |  |  |  |
| Precentral Gyrus | 23 | 50 | -6 | 34 | 10.38 | Precentral Gyrus (40%) | Area 4p (73%) |  |  |  |  |
| Putamen |  | 30 | -8 | 2 | 28.16 | Putamen (100%) |  | Parietal (50%) |  | Motor | Motor |
| Rolandic Operculum | 32 | 38 | -26 | 18 | 10.15 | Parietal Operculum (58%) | OP2 (38%) | IPLA (62.5%) |  |  |  |
| Supramarginal gyrus | 591 | 58 | -30 | 30 | 13.32 | Supramarginal Gyrus, posterior division (35%) | PFcm (34%) | IPLA,B (37.5%) |  |  |  |
| Precuneus | 29 | 4 | -36 | 48 | 9.77 | Precuneus (38%) | Area 5Ci (43%) |  |  |  |  |
| Superior Parietal Lobule | 458 | 30 | -46 | 48 | 12.54 | Superior Parietal Lobule (44%) | Area 7PC (32%) | SPLA (75%) |  |  |  |
| Fusiform Gyrus | 87 | 38 | -58 | -20 | 12.61 | Temporal Occipital Fusiform Gyrus (51%) | FG2 (39%) |  |  |  |  |
| Superior Parietal Lobule | 21 | 22 | -62 | 68 | 10.29 | Lateral Occipital Cortex, superior division (53%) | Area 7A (80%) | SPLB,C,D (25%) |  |  |  |
| Cerebellum | 127 | 12 | -66 | -20 | 13.21 |  |  |  | Lobule HVI (93%) |  |  |

**Supplemental Table 7**: Regions showing significantly greater connectivity with unassigned voxels compared to assigned using the conjunction analysis (voxel-level Z>3.1, cluster-level p<0.05 FDR). K indicates cluster size in voxel number (each voxel is 2x2x2mm). X, Y, Z co-ordinates are in MNI space. Cortical localization used the Harvard-Oxford probabilistic atlas, Jülich atlas, and connectivity-based parcellation atlases provided in FSLEYES. Striatal localization used the atlases of Pauli et al (Pauli et al., 2016), Choi et al (Choi et al., 2012), and Tziortzi et al (Tziortzi et al., 2014). Cerebellar localization used the probabilistic atlas of Diedrichsen et al (Diedrichsen et al., 2009).

| **Label** | **K** | **x** | **y** | **z** | **F** | **Harvard Oxford** | **Juelich** | **Connectivity** | **Diedrichsen** | **Pauli** | **Choi** |
| --- | --- | --- | --- | --- | --- | --- | --- | --- | --- | --- | --- |
| Frontal Pole | 43 | 32 | 66 | 0 | 2.03 | Frontal Pole (46%) | FP1 (68%) | FPl |  |  |  |
| Frontal Pole | 39 | 28 | 62 | -12 | 2.04 | Frontal Pole (74%) | FP1 (70%) | FPl (81.3%) |  |  |  |
| Frontal Pole | 66 | 40 | 56 | 14 | 1.89 | Frontal Pole (83%) |  | Area 46 (38.7%) |  |  |  |
| Putamen | 290 | 24 | 12 | -2 | 8.75 | Putamen (100%) |  | Executive (84.6%) |  | Social/Language | Ventral Attention |
| Caudate Nucleus | 383 | 14 | 10 | 8 | 7.42 | Caudate (51.9%) |  | Executive (34.6%) |  | Action Value | FPN |
| Thalamus | 383 | 16 | -16 | 18 | 3.06 | Thalamus (58.9%) |  | Prefrontal (45%) |  |  |  |
| Cerebellum | 422 | 30 | -64 | -26 | 3.06 |  |  |  | HVI (90%) |  |  |
| Cerebellum | 325 | 28 | -72 | -48 | 2.7 |  |  |  | HVIIb (60%); Crus II (44%) |  |  |

**Supplemental Table 8**: Regions showing significant connectivity (voxel-level Z>3.1, cluster-level p<0.05 FDR) with the macaque-mouse mCP map. K indicates cluster size in voxel number (each voxel is 1.5x1.5x1.5mm). X, Y, Z co-ordinates are in F99/McLaren space (McLaren et al., 2009). Activations were localization used Saleem-Logothetis atlas (Saleem and Logothetis, 2007).

| **Label** | **K** | **x** | **y** | **z** | **T** | **Saleem-Logothetis** |
| --- | --- | --- | --- | --- | --- | --- |
| Superior Frontal Gyrus | 15 | 4 | 24 | 17 | 8.47 | 9m |
| Anterior Cingulate Cortex | 1379 | 2 | 10 | 12 | 7.97 | Area 24 |
| Caudate Nucleus | 1379 | 7 | 9 | 5 | 42.52 |  |
| Precentral Gyrus | 1379 | 16 | 7 | 9 | 8.52 | Area 45b |
| Superior Frontal Gyrus | 37 | 11 | 6 | 17 | 5.98 | F2 (6DR/6DC) |
| Precentral Gyrus | 1379 | 23 | 2 | 2 | 11.88 | F5 |
| lateral amygdala | 1379 | 14 | 0 | -13 | 12.19 |  |
| Caudate Nucleus | 1379 | 5 | -1 | 9 | 12.08 |  |
| Middle Temporal Gyrus | 1379 | 20 | -6 | -10 | 8.22 | IPa (sts fundus) |
| Middle Temporal Gyrus | 1379 | 20 | -15 | -4 | 9.51 | IPa (sts fundus) |
| Posterior Cingulate gyrus | 29 | 2 | -19 | 14 | 7.61 | 23b |
| Supramarginal gyrus | 13 | 16 | -22 | 21 | 9.67 | LIPd |
| Cuneus | 13 | 16 | -27 | 3 | 8.96 | V1 |
| angular gyrus | 20 | 11 | -37 | 18 | 5.73 | V2 |
| Cuneus | 16 | 2 | -37 | 15 | 5.49 | V1 |

**Supplemental Table 9**: Regions showing significant connectivity (voxel-level Z>3.1, cluster-level p<0.05 FDR) with the macaque-mouse NAcc map. K indicates cluster size in voxel number (each voxel is 1.5x1.5x1.5mm). X, Y, Z co-ordinates are in F99/McLaren space (McLaren et al., 2009). Activations were localization used Saleem-Logothetis atlas (Saleem and Logothetis, 2007).

| **Label** | **K** | **x** | **y** | **z** | **T** | **Saleem-Logothetis** |
| --- | --- | --- | --- | --- | --- | --- |
| Anterior Cingulate Cortex | 1153 | 2 | 18 | 11 | 5.41 | Area 24 |
| Nucleus Accumbens | 1153 | 2 | 7 | 3 | 66.84 |  |
| Precentral Gyrus | 1153 | 19 | 7 | 9 | 7.95 | Area 44 |
| Precentral Gyrus | 1153 | 23 | 2 | 2 | 11.88 | F5 |
| Precentral Gyrus | 1153 | 25 | 2 | 9 | 9.81 | F4 |
| Amygdala | 1153 | 13 | -1 | 15 | 14.94 |  |
| Middle Temporal Gyrus | 1153 | 20 | -6 | -10 | 8.22 | IPa (sts fundus) |
| Hipocampus | 1153 | 17 | -16 | -6 | 14.74 | Dentate Gyrus |
| Superior Temporal Gyrus | 16 | 14 | -25 | 15 | 5.87 | Medial Superior Temporal Area |
| Angular Gyrus | 17 | 11 | -36 | 18 | 6.34 | V2 |

**Supplemental Table 10**: Regions showing significant connectivity (voxel-level Z>3.1, cluster-level p<0.05 FDR) with the macaque-mouse lCP map. K indicates cluster size in voxel number (each voxel is 1.5x1.5x1.5mm). X, Y, Z co-ordinates are in F99/McLaren space (McLaren et al., 2009). Activations were localization used Saleem-Logothetis atlas (Saleem and Logothetis, 2007).

| **Label** | **K** | **x** | **y** | **z** | **T** | **Saleem-Logothetis** |
| --- | --- | --- | --- | --- | --- | --- |
| Anterior Cingulate Cortex | 23 | 4 | 12 | 14 | 5.8 | Area 24 |
| Superior Frontal Gyrus | 191 | 4 | 6 | 23 | 5.72 | F2 (6DR/6DC) |
| Caudate Nucleus | 1007 | 8 | 3 | 8 | 14.36 |  |
| Precentral Gyrus | 1007 | 16 | 3 | 9 | 6.56 | F5 (6Va,b) |
| Putamen | 1007 | 17 | -3 | 3 | 25.26 |  |
| Postcentral gyrus | 14 | 16 | -9 | 14 | 5.26 | AIP |
| Posterior Cingulate gyrus | 191 | 5 | -12 | 14 | 8.05 | Area 23 |
| Postcentral gyrus | 30 | 13 | -13 | 20 | 6.52 | S1-2 |
| Supramarginal gyrus | 46 | 17 | -16 | 15 | 5.72 | LIPv |
| Posterior Cingulate gyrus | 191 | 2 | -19 | 14 | 8.04 | Area 31 |
| Supramarginal gyrus | 46 | 14 | -22 | 20 | 7.6 | LIPd |

**Supplemental Table 11**: Regions showing significantly greater connectivity with unassigned voxels compared to assigned using the conjunction analysis (voxel-level Z>3.1, cluster-level p<0.05 FDR). K indicates cluster size in voxel number (each voxel is 1.5x1.5x1.5mm). X, Y, Z co-ordinates are in F99/McLaren space (McLaren et al., 2009). Activations were localization used Saleem-Logothetis atlas (Saleem and Logothetis, 2007).

| **Label** | **K** | **x** | **y** | **z** | **F** | **Saleem-Logothetis** |
| --- | --- | --- | --- | --- | --- | --- |
| Middle Frontal Gyrus | 31 | 14 | 13 | 15 | 2.02 | Area 46d |
| Precentral gyrus | 255 | 17 | 7 | 5 | 2.29 | Gustatory cortex |
| Caudate Nucleus | 255 | 7 | 4 | 6 | 11.74 |  |
| Superior Frontal gyrus | 31 | 13 | 4 | 18 | 2.16 | F2 (6DR/6DC) |
| Caudate Nucleus | 255 | 5 | -6 | 8 | 8.97 |  |
| Putamen | 12 | 17 | -7 | -3 | 3.03 |  |
| Superior Temporal Gyrus | 28 | 13 | -25 | 15 | 2.29 | Medial Superior Temporal Area |
| Supremarginal gyrus | 44 | 10 | -31 | 21 | 1.63 | Area 7A |
| Superior Parietal lobule | 44 | 5 | -36 | 14 | 2.54 | V1 |

**Supplemental Table 12**: Regions showing significant connectivity (voxel-level Z>3.1, cluster-level p<0.05 FDR) with the human-macaque caudate map. K indicates cluster size in voxel number (each voxel is 2x2x2mm). X, Y, Z co-ordinates are in MNI space. Cortical localization used the Harvard-Oxford probabilistic atlas, Jülich atlas (*Area 6 probabilities were taken from the Jülich atlas in FSLEYES), and connectivity-based parcellation atlases provided in FSLEYES. Striatal localization used the atlases of Pauli et al (Pauli et al., 2016), Choi et al (Choi et al., 2012), and Tziortzi et al (Tziortzi et al., 2014). Cerebellar localization used the probabilistic atlas of Diedrichsen et al (Diedrichsen et al., 2009).

| **Label** | **K** | **x** | **y** | **z** | **T** | **Harvard Oxford** | **Juelich** | **Connectivity** | **Diedrichsen** | **Pauli** | **Choi** |
| --- | --- | --- | --- | --- | --- | --- | --- | --- | --- | --- | --- |
| Middle Frontal Gyrus | 888 | 38 | 44 | 26 | 11.72 | Frontal Pole (83%) |  | Area 46 (100%) |  |  |  |
| Middle Cingulate Cortex | 947 | 10 | 26 | 30 | 10.29 | Paracingulate Gyrus (47%) |  | RCZa (76.3%) |  |  |  |
| Inferior Frontal Gyrus pars Triangularis | 1068 | 40 | 24 | 6 | 11.02 | Frontal Operculum (52%) |  |  |  |  |  |
| Caudate Nucleus | 1870 | 8 | 14 | 0 | 28.3 | Caudate (85.8%) |  | Executive (69.2%) |  | Action Value | DMN |
| Middle Frontal Gyrus | 276 | 42 | 4 | 60 | 10.42 | Middle Frontal Gyrus (47%) | Area 6 (12%)* |  |  |  |  |
| Putamen | 1870 | 24 | 4 | 6 | 32.42 | Putamen (100%) |  | Executive (65.4%) |  | Social/Language | Ventral Attention |
| Superior Temporal Gyrus | 20 | 54 | -18 | 6 | 7.84 | Heschl's gyrus (25%) | Area TE (32%) |  |  |  |  |
| Posterior Cingulate Cortex | 80 | 2 | -34 | 48 | 7.34 | Cingulate Cortex, posterior (44%) | Area 5Ci (31%) | CCZ (29.2%) |  |  |  |
| Inferior Parietal Lobule | 9.14 | 42 | -50 | 50 | 9.14 | Angular Gyrus (35%) | hIP3 (45%) | IPLD (50%) |  |  |  |
| Cerebellum | 478 | 32 | -70 | -22 | 10.81 |  |  |  | HVI (58%) |  |  |
| Precuneus | 28 | 2 | -74 | 52 | 7.34 | Precuneus (36%) | Area 7p (29%) | SPLE (50%) |  |  |  |
| Cerebellum | 35 | 24 | -76 | -44 | 8.4 |  |  |  | Crus II (46%) |  |  |
| Occipital Lobe | 99 | 38 | -92 | -14 | 9.29 | Occipital Pole (14%) | V3 (63%) |  |  |  |  |
| Occipital Lobe | 66 | 10 | -96 | -6 | 8.47 | Occipital Pole (61%) | V1 (76%) |  |  |  |  |

**Supplemental Table 13**: Regions showing significant connectivity (voxel-level Z>3.1, cluster-level p<0.05 FDR) with the human-macaque NAcc map. K indicates cluster size in voxel number (each voxel is 2x2x2mm). X, Y, Z co-ordinates are in MNI space. Cortical localization used the Harvard-Oxford probabilistic atlas, Jülich atlas, and connectivity-based parcellation atlases provided in FSLEYES. Striatal localization used the atlases of Pauli et al (Pauli et al., 2016), Choi et al (Choi et al., 2012), and Tziortzi et al (Tziortzi et al., 2014). Cerebellar localization used the probabilistic atlas of Diedrichsen et al (Diedrichsen et al., 2009).

|  | **K** | **x** | **y** | **z** | **T** | **Harvard Oxford** | **Juelich** | **Connectivity** | **Diedrichsen** | **Pauli** | **Choi** |
| --- | --- | --- | --- | --- | --- | --- | --- | --- | --- | --- | --- |
| Anterior Cingulate Cortex | 113 | 10 | 42 | 6 | 9.23 | Cingulate Cortex, anterior (49%) |  | Area 32pl (81.8%) |  |  |  |
| Nucleus Accumbens | 1140 | 10 | 12 | -6 | 65.23 | Nacc (92.3%) |  | Limbic (65.4%) |  | Stimulus Value | Limbic |
| Cerebellum | 21 | 2 | -50 | -36 | 8.36 |  |  |  | IX (64%) |  |  |

**Supplemental Table 14**: Regions showing significant connectivity (voxel-level Z>3.1, cluster-level p<0.05 FDR) with the human-macaque putamen map. K indicates cluster size in voxel number (each voxel is 2x2x2mm). X, Y, Z co-ordinates are in MNI space. Cortical localization used the Harvard-Oxford probabilistic atlas, Jülich atlas (*Area 6 probabilities were taken from the Jülich atlas in FSLEYES), and connectivity-based parcellation atlases provided in FSLEYES. Striatal localization used the atlases of Pauli et al (Pauli et al., 2016), Choi et al (Choi et al., 2012), and Tziortzi et al (Tziortzi et al., 2014). Cerebellar localization used the probabilistic atlas of Diedrichsen et al (Diedrichsen et al., 2009).

| **Label** | **K** | **x** | **y** | **z** | **T** | **Harvard Oxford** | **Juelich** | **Connectivity** | **Diedrichsen** | **Pauli** | **Choi** |
| --- | --- | --- | --- | --- | --- | --- | --- | --- | --- | --- | --- |
| Middle Frontal Gyrus | 496 | 38 | 44 | 26 | 11.95 | Frontal Pole (83%) |  | Area 46 (100%) |  |  |  |
| Middle Frontal Gyrus | 168 | 50 | 44 | 6 | 8.61 | Frontal Pole (81%) |  |  |  |  |  |
| Middle Cingulate Cortex | 1268 | 10 | 24 | 32 | 11.23 | Paracingulate Gyrus (47%) |  | RCZa (66.1%) |  |  |  |
| Putamen | 3985 | 24 | 4 | 6 | 30.99 | Putamen (100%) |  | Executive (65.4%) |  | Social/Language | Ventral Attention |
| Middle Frontal Gyrus | 335 | 42 | 2 | 56 | 8.9 | Middle Frontal Gyrus (48%) | Area 6 (30%)* |  |  |  |  |
| Posterior Cingulate Cortex | 173 | 0 | -24 | 46 | 8.11 | Cingulate Cortex, posterior (39%) |  |  |  |  |  |
| Middle Temporal Gyrus | 35 | 52 | -26 | -2 | 7.81 | Superior Temporal Gyrus, posterior (26%) |  |  |  |  |  |
| Supramarginal gyrus | 1119 | 66 | -36 | 30 | 13.12 | Supremarginal Gyrus, posterior (45%) | Area PF (74%) | TPJa (62.5%); IPLB (37.5%) |  |  |  |
| Cerebellum | 249 | 32 | -70 | -22 | 9.36 |  |  |  | HVI (58%) |  |  |
| Occipital Lobe | 116 | 40 | -92 | -12 | 8.76 | Lateral Occipital Cortex, inferior (13%) | V3v (57%) |  |  |  |  |

**Supplemental Table 15**: Regions showing significantly greater connectivity with unassigned voxels compared to assigned using the conjunction analysis (voxel-level Z>3.1, cluster-level p<0.05 FDR). K indicates cluster size in voxel number (each voxel is 2x2x2mm). X, Y, Z co-ordinates are in MNI space. Cortical localization used the Harvard-Oxford probabilistic atlas, Jülich atlas, and connectivity-based parcellation atlases provided in FSLEYES. Striatal localization used the atlases of Pauli et al (Pauli et al., 2016), Choi et al (Choi et al., 2012), and Tziortzi et al (Tziortzi et al., 2014). Cerebellar localization used the probabilistic atlas of Diedrichsen et al (Diedrichsen et al., 2009).

| **Label** | **K** | **x** | **y** | **z** | **T** | **Harvard Oxford** | **Juelich** | **Connectivity** | **Diedrichsen** | **Pauli** | **Choi** |
| --- | --- | --- | --- | --- | --- | --- | --- | --- | --- | --- | --- |
| Frontal Pole | 363 | 30 | 54 | -6 | 3.04 | Frontal Pole (54%) | Fp1 (70%) | Area FPl (96%) |  |  |  |
| Caudate Nucleus | 652 | 18 | 20 | 10 | 7.25 | Caudate (80.3%) |  | Executive (53.8%) |  | Action Value | FPN |
| Thalamus | 652 | 2 | -20 | 12 | 5.06 | Thalamus (30.5%) |  | Temporal Thalamus (33%) |  |  |  |
| Cerebellum | 106 | 28 | -32 | -36 | 2.61 |  |  |  | HV (54%) |  |  |
| Precuneus | 64 | 14 | -66 | 44 | 3.11 | Precuneus (43%) |  | SPLD (62.5%) |  |  |  |
| Cerebellum | 793 | 42 | -70 | -26 | 3.37 |  |  |  | Crus I (99%) |  |  |
| Occiptal Lobe | 1029 | 24 | -96 | 0 | 3.4 | Occipital Pole (50%) | V1 (39%) |  |  |  |  |

# References

Amunts K, Kedo O, Kindler M, Pieperhoff P, Mohlberg H, Shah NJ, Habel U, Schneider F, Zilles K. 2005. Cytoarchitectonic mapping of the human amygdala, hippocampal region and entorhinal cortex: intersubject variability and probability maps. *Anat Embryol* **210**:343–352. doi:10.1007/s00429-005-0025-5

Balleine BW, apos Doherty JP. 2009. Human and Rodent Homologies in Action Control: Corticostriatal Determinants of Goal-Directed and Habitual Action. *Neuropsychopharmacol* **35**:48 69. doi:10.1038/npp.2009.131

Choi E, Yeo TB, Buckner RL. 2012. The organization of the human striatum estimated by intrinsic functional connectivity. *J Neurophysiol* **108**:2242 2263. doi:10.1152/jn.00270.2012

Diedrichsen J, Balsters JH, Flavell J, Cussans E, Ramnani N. 2009. A probabilistic MR atlas of the human cerebellum. *Neuroimage* **46**:39–46. doi:10.1016/j.neuroimage.2009.01.045

Eickhoff SB, Jbabdi S, Caspers S, Laird AR, Fox PT, Zilles K, Behrens TE. 2010. Anatomical and functional connectivity of cytoarchitectonic areas within the human parietal operculum. *J Neurosci* **30**:6409 6421. doi:10.1523/jneurosci.5664-09.2010

Franklin K, Paxinos G. 2019. Paxinos and Franklin’s the mouse brain in stereotaxic coordinates.

Grandjean J, Canella C, Anckaerts C, Ayrancı G, Bougacha S, Bienert T, Buehlmann D, Coletta L, Gallino D, Gass N, Garin CM, Nadkarni N, Hübner N, Karatas M, Komaki Y, Kreitz S, Mandino F, Mechling AE, Sato C, Sauer K, Shah D, Strobelt S, Takata N, Wank I, Wu T, Yahata N, Yeow L, Yee Y, Aoki I, Chakravarty MM, Chang W-T, Dhenain M, von Elverfeldt D, Harsan L-A, Hess A, Jiang T, Keliris GA, Lerch JP, Meyer-Lindenberg A, Okano H, Rudin M, Sartorius A, der Linden A, Verhoye M, Weber-Fahr W, Wenderoth N, Zerbi V, Gozzi A. 2019. Common functional networks in the mouse brain revealed by multi-centre resting-state fMRI analysis. *Neuroimage* 116278. doi:10.1016/j.neuroimage.2019.116278

Heilbronner SR, Rodriguez-Romaguera J, Quirk GJ, Groenewegen HJ, Haber SN. 2016. Circuit-Based Corticostriatal Homologies Between Rat and Primate. *Biol Psychiat* **80**:509–521. doi:10.1016/j.biopsych.2016.05.012

Margulies DS, Vincent JL, Kelly C, Lohmann G, Uddin LQ, Biswal BB, Villringer A, Castellanos XF, Milham MP, Petrides M. 2009. Precuneus shares intrinsic functional architecture in humans and monkeys. *Proc National Acad Sci* **106**:20069–20074. doi:10.1073/pnas.0905314106

Mars RB, Sallet J, Neubert F-X, Rushworth MF. 2013. Connectivity profiles reveal the relationship between brain areas for social cognition in human and monkey temporoparietal cortex. *Proc National Acad Sci* **110**:10806 10811. doi:10.1073/pnas.1302956110

Mayka MA, Corcos DM, Leurgans SE, Vaillancourt DE. 2006. Three-dimensional locations and boundaries of motor and premotor cortices as defined by functional brain imaging: A meta-analysis. *Neuroimage* **31**:1453 1474. doi:10.1016/j.neuroimage.2006.02.004

McLaren DG, Kosmatka KJ, Oakes TR, Kroenke CD, Kohama SG, Matochik JA, Ingram DK, Johnson SC. 2009. A population-average MRI-based atlas collection of the rhesus macaque. *Neuroimage* **45**:52–59. doi:10.1016/j.neuroimage.2008.10.058

Neubert F-X, Mars RB, Sallet J, Rushworth MF. 2015. Connectivity reveals relationship of brain areas for reward-guided learning and decision making in human and monkey frontal cortex. *Proc National Acad Sci* **112**:E2695–E2704. doi:10.1073/pnas.1410767112

Neubert F-X, Mars RB, Thomas AG, Sallet J, Rushworth M. 2014. Comparison of Human Ventral Frontal Cortex Areas for Cognitive Control and Language with Areas in Monkey Frontal Cortex. *Neuron* **81**:700–713. doi:10.1016/j.neuron.2013.11.012

Pauli WM, O’Reilly RC, Yarkoni T, Wager TD. 2016. Regional specialization within the human striatum for diverse psychological functions. *Proc National Acad Sci* **113**:1907 1912. doi:10.1073/pnas.1507610113

Paxinos G, X-F H, Petrides M, Toga A. 2009. The rhesus monkey brain in stereotaxic coordinates.

Saleem K, Logothetis N. 2007. A Combined MRI and Histology Atlas of the Rhesus Monkey Brain in Stereotaxic Coordinates.

Sallet J, Mars R, Noonan M, Neubert F, Jbabdi S, O’Reilly J, Filippini N, Thomas A, Rushworth M. 2013. The Organization of Dorsal Frontal Cortex in Humans and Macaques. *J Neurosci* **33**:12255 12274. doi:10.1523/jneurosci.5108-12.2013

Strange BA, Witter MP, Lein ES, Moser EI. 2014. Functional organization of the hippocampal longitudinal axis. *Nat Rev Neurosci* **15**:655 669. doi:10.1038/nrn3785

Tziortzi AC, Haber SN, Searle GE, Tsoumpas C, Long CJ, Shotbolt P, Douaud G, Jbabdi S, Behrens TE, Rabiner EA, Jenkinson M, Gunn RN. 2014. Connectivity-based functional analysis of dopamine release in the striatum using diffusion-weighted MRI and positron emission tomography. *Cereb Cortex* **24**:1165 1177. doi:10.1093/cercor/bhs397

Vogt BA, Paxinos G. 2014. Cytoarchitecture of mouse and rat cingulate cortex with human homologies. *Brain Struct Funct* **219**:185–192. doi:10.1007/s00429-012-0493-3

Wise SP. 2008. Forward frontal fields: phylogeny and fundamental function. *Trends Neurosci* **31**:599–608. doi:10.1016/j.tins.2008.08.008

Zerbi V, Grandjean J, Rudin M, Wenderoth N. 2015. Mapping the mouse brain with rs-fMRI: An optimized pipeline for functional network identification. *Neuroimage* **123**:11–21. doi:10.1016/j.neuroimage.2015.07.090
